# The impact of iron store on red blood cell transfusion: a multicentre prospective cohort study in cardiac surgery

**DOI:** 10.1186/s12871-022-01616-6

**Published:** 2022-03-21

**Authors:** Baptiste Gaudriot, Jean-Ferreol Oilleau, Thomas Kerforne, Claude Ecoffey, Olivier Huet, Alexandre Mansour, Jean-Philippe Verhoye, Nicolas Massart, Nicolas Nesseler, for the AtlanRea Study Group, Baptiste Gaudriot, Jean-Ferreol Oilleau, Thomas Kerforne, Claude Ecoffey, Olivier Huet, Alexandre Mansour, Nicolas Massart, Nicolas Nesseler

**Affiliations:** 1 Department of Anaesthesia and Critical Care, University Hospital of Rennes, 35000 Rennes, France; 2 Service d’Anesthésie-Réanimation CTCV, Centre Cardio-Pneumologique, Hôpital Pontchaillou, 2 rue Henri Le Guilloux, 35033, Cedex 9 Rennes, France; 3 Department of Anaesthesia and Critical Care, Brest University Hospital, 29000 Brest, France; 4 Department of Anaesthesia and Critical Care, INSERM U-1082, Poitiers University Hospital, 86000 Poitiers, France; 5 Univ Rennes, CHU de Rennes, 35000 Rennes, France; 6 Brest University, 29000 Brest, France; 7 Thoracic and Cardiovascular Surgery, University Hospital of Rennes, Univ Rennes 1, 35000 Rennes, France; 8 Intensive Care Unit, Saint-Brieuc Hospital, 22000 Saint-Brieuc, France; 9 Univ Rennes, CHU de Rennes, Inra, Inserm, Institut NUMECAN – UMR_A 1341, UMR_S 1241, F-35000 Rennes, France

**Keywords:** Cardiac surgery, Cardiopulmonary bypass, Iron deficiency, Anaemia, Transfusion, Patient blood management

## Abstract

**Background:**

Anaemia is common prior to cardiac surgery and contributes to perioperative morbidity. Iron deficiency is the main cause of anaemia but its impact remains controversial in the surgical setting. We aimed to estimate the impact of iron deficiency on in-hospital perioperative red blood cell transfusion for patients undergoing elective and urgent cardiac surgery. Secondary objectives were to identify risk factors associated with in-hospital red blood cell transfusion.

**Methods:**

We conducted a prospective multicentre observational study in three university hospitals performing cardiac surgery. We determined iron status prior to surgery and collected all transfusion data to compare iron-deficient and iron-replete patients during hospital stay. We performed a multivariable logistic regression to compare transfusion among groups.

**Results:**

Five hundred and two patients were included. A trend of low haemoglobin levels associated with iron deficiency persisted until discharge. Red blood cell transfusion was significantly higher in the group of iron deficient patients during surgery (22% vs 13%, *p* = 0.017), however the incidence during the whole hospital stay was 31% in the iron-deficient group, not significantly different with the non-deficient group (26%, *p* = 0.28). Iron deficiency was not independently associated with in-hospital red blood cell transfusion (adjusted OR = 0.85 [0.53–1.36], *p* = 0.49).

**Conclusions:**

In-hospital red blood cell transfusion was not significantly higher in iron-deficient patients and iron deficiency was not associated with in-hospital red blood cell transfusion in patients undergoing elective and urgent cardiac surgery. Iron deficiency was the main cause of anaemia and anaemia was a strong driver of red blood cell transfusion. Further studies should identify sub-population of iron-deficient patients which may benefit from preoperative iron deficiency management and explore the long-term impact of lower haemoglobin levels at discharge in the iron deficient population.

## Background

Iron deficiency (ID) remains the main cause of anaemia worldwide [1]. ID can present as multiple clinical and biological signs, from fatigue and impaired physical capacity to iron deficiency anaemia (IDA) at a later stage. Absolute ID (AID), referring to the reduction of iron stores, is commonly defined by a serum ferritin < 30 ng ml^−1^. Functional ID (FID), referring to misuse of iron from stores, has to be proven by a low transferrin saturation (Tsat) level that indicates an insufficient iron supply for normal erythropoiesis [2].

The international consensus statement on the perioperative management of anaemia and iron deficiency recommends to screen and to investigate anaemia before all surgical procedures when a moderate-to-high blood loss is expected [3]. The experts suggest that ID and/or inadequate iron stores before such major surgeries (ferritin level below 100 ng ml^−1^, especially associated with a Tsat < 20%) should be treated as early as possible while surgery should be postponed if mitigation cannot be achieved. Similarly, among heart failure patients, the European Society of Cardiology advises to consider iron supplementation in symptomatic patients with reduced ejection fraction and ID (serum ferritin < 100 μg l^−1^, or ferritin between 100–299 μg l^−1^ and Tsat < 20%) [4].

We previously observed that 31% of patients presented ID prior to elective cardiac surgery [5], consistent with available reports [6, 7]. Both anaemia and red blood cell (RBC) transfusions are associated with poor outcome in elective cardiac surgery [8–11]. No recommendations are currently available for ID management prior to cardiac surgery (irrespective of anaemia) and a better understanding of its actual impact on perioperative outcomes is crucial to design further studies for preventive strategies. ID and IDA are modifiable risk factors for RBC transfusion, but their association with transfusion and outcomes remains controversial in cardiac surgery [12–14].

Therefore, we designed a multicentre observational study in order to assess the impact of ID among patients undergoing elective and urgent non-emergency cardiac surgery. The primary aim was to evaluate the impact of ID on perioperative RBC transfusions over the course of the entire hospital stay, and secondary objectives were to identify risk factors for in-hospital RBC transfusions, and evaluate the association of ID with postoperative bleeding and outcomes.

## Methods

### Patient population

This prospective observational study has been conducted in 3 university hospitals in France between November 2016 and October 2017. All patients presenting for elective or urgent cardiac surgery with routine determination of pre-operative iron status were eligible. Exclusion criteria were: patients younger than 18 years of age or legally protected adults, endovascular procedures (trans-aortic valve replacement and all percutaneous techniques), surgeries on the descending aorta, emergency (< 24 h delay) and salvage surgeries, and patients affected by an iron overload disease. We used the Strengthening the Reporting of Observational Studies in Epidemiology (STROBE) tools [15] to conduct and report this work.

The study protocol was approved by the ethics committee of Rennes university hospital (notice n° 16.121 of October 7, 2016, Dr Morel, M.D.). All participants received oral and written information. Written consent was waived due to the observational design of this study and the provision of standard care to all patients.

### Study endpoint and definitions

We hypothesised that ID would increase perioperative RBC transfusions, defined as RBC transfusion during the whole hospital stay. Iron deficiency was defined according to the current guidelines by a ferritin level < 100 ng ml^−1^, or a ferritin range 100–300 ng ml^−1^ in association with Tsat < 20% [3, 4]. Anaemia was defined according to World Health Organization criteria: haemoglobin (Hb) concentration below 13 g dl^−1^ in men and below 12. g dl^−1^ in women.

### Study procedures and data collection

Comorbidities and baseline characteristics were recorded at patient admission from routine pre-operative evaluation. As systematically done for all cardiac surgery patients in our institutions, biological tests (Hb level, platelet count and ionogram, including serum creatinine) were collected pre-operatively. Since all centres were in a transition toward a better management of preoperative anaemia, they all recently associated a routine preoperative determination of the iron status including serum ferritin, transferrin and iron levels. Transferrin saturation was calculated as follows: Tsat (%) = serum iron (μmol l^−1^) (serum transferrin (g l^−1^) × 25)^−1^. Preoperative treatments for anaemia or iron deficiency were not yet available in our centres during the study period though. To prevent blood loss, every patient received an antifibrinolytic agent (namely tranexamic acid 50–75 mg.kg^−1^) and intraoperative cell salvaging. Decision to transfuse RBC was conducted with a restrictive transfusion policy. During cardiopulmonary bypass (CPB), a multiparametric evaluation including haematocrit and tissue oxygenation aimed to initiate RBC transfusion if haemoglobin was < 6.0 g dl^−1^, with an acceptable haematocrit value established between 21 and 24% to maintain the DO2 above 273 ml min^−1^ m^−2^, as recommended [16, 17]. During off-pump surgeries, intraoperative post-CPB period, and for the postoperative period of every surgery, transfusion was based on the French recommendations. Transfusion thresholds were below 7 g dl^−1^ for patients with no particular history, below 8 to 9 g dl^−1^ for patients with a previous cardiovascular history, and below 10 g dl^−1^ for vulnerable patients with acute coronary insufficiency, cardiac failure, or not able to tolerate lower levels of haemoglobin [18]. Apart from these indications and if the haemoglobin level was found too low to achieve the postoperative rehabilitation, anaemic patients could receive postoperative intravenous iron.

### Sample size calculation

The number of patients needed to be included was calculated based on our previous work [5] which included elective cardiac surgery cases. RBC transfusion incidence was 30% for patients with ID and 22% for patients without, with an ID prevalence of 31%. In the present study we included both elective and urgent cardiac surgeries. We therefore expected a higher incidence of RBC transfusion [19] and a higher prevalence of ID [20]. RBC transfusion rates of 40% and 25% were estimated in the ID and in the non-ID groups respectively. Considering that, 406 patients were needed in order to observe such a difference with an α risk of 5% and β at 10%.

### Statistical analysis

Quantitative variables are presented as the mean ± standard deviation or as median (Q1-Q3), as appropriate. Groups were compared using the non-parametric test of Wilcoxon. Categorical variables are presented as the number of patients by percentage: n (%) and we compared the groups using the χ2 test or Fisher’s exact test, depending on the number of patients.

Univariable and multivariable logistic regression models were used to study risk factors. For the multivariable analysis, all variables associated with RBC transfusion with a p-value < 0.2 in univariate analysis were included in the first multivariable model, then a backward stepwise selection was applied to produce the final model containing independent predictors at p < 0.1 only. To account for centre-related effect, multivariable logistic regression was stratified by centre with a fixed effect conditional logistic regression model. Results are presented as adjusted odd ratio (OR) (95% CI). All tests were two-sided and the difference was considered significant for p < 0.05. Statistical analyses were performed using R® software, version 3.5.3.

## Results

### Descriptive analysis

**Fig. 1:**
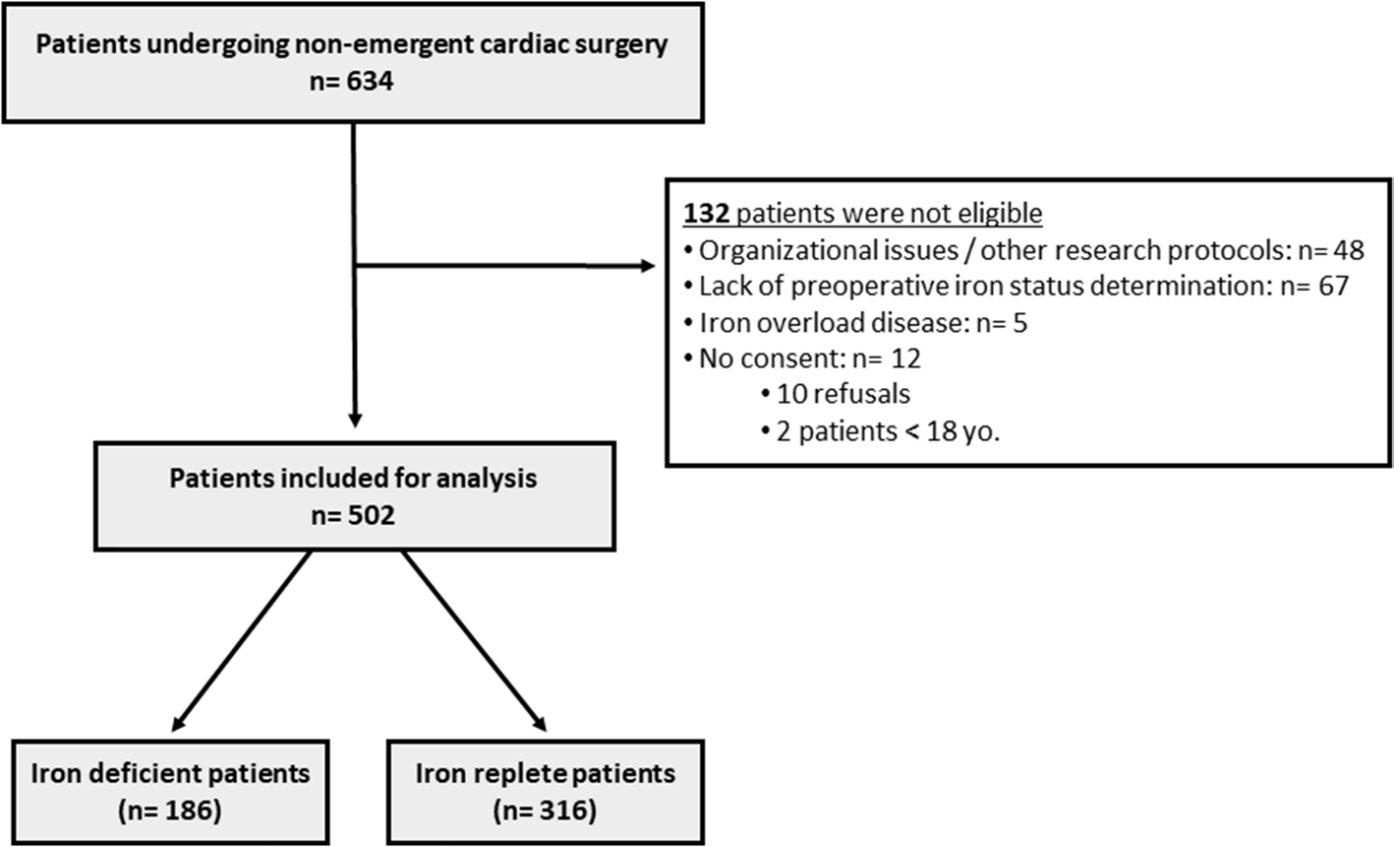
Study flowchart

Five hundred and two patients were included during the study period (312 in centre A, 172 in centre B, and 18 in centre C), of whom 186 (37%) had ID. The flowchart of the study is shown in Fig. 1. Most of the surgeries were coronary bypasses (41%) and isolated valves (37%) electively performed under CPB. Anaemia was common among patients with ID (29%). Study population, pre-operative biological status and surgery characteristics are presented in Table 1.

**Table 1:** Population characteristics

	**Population (*n* = 502)**	**Patients with iron deficiency (*n* = 186)**	**Patients with RBC transfusion (*n* = 141)**
**Patients characteristics**			
** Age** (years)^a^	67 ± 11 || 69(61–76)	67 ± 12 || 69(61–76)	69 ± 11|| 70 (64–77)
** Sex** (male)^b^	364 (73%)	108 (58%)	75 (53%)
** BMI** (kg m^−2^)^a^	27 ± 4 || 26(24–29)	27 ± 5 || 26(24–30)	28 ± 4 || 26 (23–29)
** SAPS II**^a^	28 ± 10 || 27(21–33)	28 ± 10 || 27(21–33)	31 (25–37)
**ASA score**^b^			
** I**	1 (0%)	1 (1%)	0
** II**	7 (1%)	2 (1%)	1 (1%)
** III**	435 (87%)	160 (86%)	117 (83%)
** IV**	59 (12%)	23 (12%)	23 (16%)
** Smoking**^b^	227 (45%)	73 (39%)	56 (40%)
** Hypertension**^b^	317 (63%)	121 (65%)	99 (70%)
** Dyslipidaemia**^b^	234 (47%)	83 (45%)	63 (45%)
** Arteritis**^b^	46 (9%)	23 (12%)	17 (12%)
** Angina**^b^	155 (31%)	51 (27%)	41 (29%)
** Obstructive lung disease**^b^	58 (12%)	26 (14%)	11 (8%)
** Diabetes**^b^	96 (19%)	44 (23%)	25 (18%)
** CRF**^b^	63 (13%)	24 (13%)	25 (18%)
** Serum creatinine** (μmol l^−1^)^a^	88 ± 27 || 83(72–96)	83 ± 24 || 80(65–96)	83 ± 27 || 80 (67–103)
** Atrial fibrillation**^b^	59 (12%)	25 (13%)	21 (15%)
**NYHA class**^b^			
** 1**	95 (19%)	27 (14%)	19 (13%)
** 2**	289 (58%)	110 (59%)	76 (54%)
** 3**	111 (22%)	46 (25%)	44 (31%)
** 4**	7 (1%)	3 (2%)	3 (2%)
** LVEF** (%)^a^	59 ± 11 || 60(55–66)	59 ± 12 || 60(55–66)	60 ± 11 || 59 (50–65)
** Pulmonary hypertension**^b^	62 (12%)	21 (11%)	24 (17%)
** Active antiplatelet therapy**^b^	271 (54%)	92 (49%)	79 (56%)
**Pre-operative biological data**			
** Pre-operative Hb** (g dl^−1^)^a^	14 ± 4 || 14 [13,14,15]	14 ± 5 || 14 [13,14,15]	13 ± 4 || 13 (12–14]
** Pre-operative anaemia**^b^	79 (16%)	16 (29%)	33 (28%)
** Serum ferritin** (ng ml^−1^)^a^	191 ± 256 || 190 (93–316)	49 ± 26 || 70 [42–105]	191 ± 331 || 190 (95–308)
** Transferrin saturation** (%)^a^	25 ± 13 || 25 (19–33)	16 ± 5 || 18 [15–26]	22 ± 13 || 23 (18–29)
** Iron Deficiency**	198 (38%)	-	61 (43%)
**Surgery characteristics**			
** Urgency of surgery**^b c^			
** Elective**	451 (90%)	168 (90%)	121 (86%)
** Urgent**	51 (10%)	18 (10%)	20 (14%)
** Surgery**^b^			
** Isolated Valve**	186 (37%)	70 (38%)	48 (40%)
** Coronary bypass**	208 (41%)	71 (38%)	50 (35%)
** Combined surgery**^d^	83 (17%)	36 (19%)	32 (24%)
** Aortic surgery**	20 (4%)	6 (3%)	11 (8%)
** Other**	5 (1%)	3 (2%)	0
** Reoperation**^b^	14 (3%)	6 (3%)	8 (6%)

*ASA* American Society of Anesthesiologists, *BMI* body mass index, *CPB* cardiopulmonary bypass, *CRF* chronic renal failure defined by a glomerular filtration rate < 60 ml min^−1^ 1.73 m^−2^, *Hb* haemoglobin, *LVEF* left ventricular ejection fraction, *NYHA* New York Heart Association classification for dyspnoea, *RBC* red blood cells, *SAPS II* Simplified Acute Physiology Score 2

^a^Mean ± standard deviation || median (Q1-Q3) ^b^Number of patients (percentage)

^c^Urgency of surgery according to the EuroSCORE II definitions. www.euroscore.org

^d^Combined surgery: surgery combining valve and coronary bypass

Patients with ID had a lower haematocrit during surgery (28% [25–31] vs 29% [26–33], *p* = 0.001) and a lower nadir of haemoglobin after surgery (9.6 g dl^−1^ [8.9–10.9] vs 10.4 g dl^−1^ [9.1–11.5], *p* < 0.001) than iron-replete patients. The trend of low haemoglobin levels associated with ID pre-operatively persisted until discharge (Table 2).

**Table 2:** Per- and postoperative outcomes

Variable	Population	Patients with ID	Patients without ID	*P*-value
	**(*n* = 502)**	**(*n* = 186)**	**(*n* = 316)**	
**Cardiopulmonary bypass**^b^	437 (87%)	157 (84%)	280 (89%)	0.22
**Circulatory arrest**^b^	16 (3%)	5 (3%)	11 (3%)	0.82
**Biological data**				
** Intraoperative**				
** Haematocrit during CPB**^a^	29 ± 5 || 29 (26–32)	28 ± 5 || 28(25–31)	29 ± 4 || 29(26–33)	**0.001**
** Postoperative (ICU)**				
** Immediate PO Hb** (g dl^−1^)^a^	11.4 ± 1.4 || 11.4(10.5–12.4)	11.3 ± 1.5 || 11.2(10.2–12.3)	11.5 ± 1.4 || 11.5(10.7–12.4)	0.08
** Minimum ICU Hb** (g dl^−1^)^a^	10.6 ± 6 || 10.1(9–11.2)	9.8 ± 1.5 || 9.6(8.9–10.9)	11 ± 7.6 || 10.4(9.1–11.5)	** < 0.001**
** Postoperative (surgical ward)**				
** Minimum Hb** (g dl^−1^)^a^	10.1 ± 1.4 || 10(9–11.1)	9.8 ± 1.3 || 9.7(8.9–10.8)	10.2 ± 1.5 || 10.1(9.1–11.3)	**0.019**
** Discharge Hb** (g dl^−1^)^a^	10.6 ± 1.4 || 10.5(9.5–11.5)	10.3 ± 1.3 || 10.2(9.3–11.3)	10.7 ± 1.4 || 10.8(9.7–11.7)	** < 0.001**
** Postoperative outcomes**				
** 24 h bleeding** (ml) *	547 ± 393 || 460(310–669)	507 ± 401 || 420(300–600)	570 ± 387 || 472(340–690)	0.08
** Haemostasis surgery within 24h**^b^	13 (3%)	5 (3%)	8 (3%)	0.92
** Postoperative infection**^b^	40 (8%)	18 (10%)	22 (7%)	0.36
** Postoperative AKF**^b^	57 (11%)	24 (13%)	33 (10%)	0.49
** Highest creatinine level** (μmol l^−1^)^a^	96 ± 68 || 83(70–101)	91 ± 50 || 80(68–101)	99 ± 77 || 83(72–101)	0.21
** Length of stay in ICU** (days)^a^	3.6 ± 3.1 || 3(2–4)	3.8 ± 3.4 || 3(2–4)	3.4 ± 2.9 || 2.5(2–4)	0.11
** Death before day 28**^b^	7 (1%)	1 (1%)	6 (2%)	0.39
** Transfusion**				
** Whole hospital stay**	*(n* = *495)*	*(n* = *185)*	*(n* = *310)*	
** All products transfusion**^b^	183 (37%)	78 (42%)	105 (33%)	0.07
** RBC** (number of unit)^a^	0.75 ± 1.6 || 0(0–1)	0.83 ± 1.9 || 0(0–2)	0.23 ± 0.5 || 0(0–0.33)	0.27
** RBC** (proportion)^b^	141 (28%)	58 (31%)	83 (26%)	0.28
** Intraoperative**				
** All products transfusion**^b^	118 (24%)	57 (31%)	61 (19%)	**0.005**
** RBC** (number of unit)^a^	0.34 ± 0.9 || 0(0–0)	0.46 ± 1.0 || 0(0–0)	0.26 ± 0.8 || 0(0–0)	**0.010**
** RBC** (proportion)^b^	81 (16%)	40 (22%)	41 (13%)	**0.017**
** Retransfusion** (ml)^a^	589 ± 350 || 522(433–715)	606 ± 387 || 541(437–737)	578 ± 327 || 514(432–710)	0.50
** ICU**				
** All products transfusion**^b^	88 (18%)	26 (14%)	62 (20%)	0.14
** RBC** (number of unit)^a^	0.36 ± 1.2 || 0(0–0)	0.27 ± 1.4 || 0(0–0)	0.41 ± 1.2 || 0(0–0)	0.14
** RBC** (proportion)^b^	73 (15%)	20 (11%)	53 (17%)	0.09
** Surgical department**	*(n* = *495)*	*(n* = *185)*	*(n* = *310)*	
** RBC** (number of unit)^a^	0.09 ± 0.4 || 0(0–0)	0.12 ± 0.5 || 0(0–0)	0.08 ± 0.4 || 0(0–0)	0.45
** RBC** (proportion)^b^	25 (5%)	11 (6%)	14 (5%)	0.59

*AKF* Acute Renal Failure, *CPB* cardiopulmonary bypass, *Hb* haemoglobin, *ICU* Intensive Care Unit, *ID* Iron Deficiency, *PO* postoperative, *RBC* red blood cell

^a^Mean ± standard deviation || median (Q1-Q3) ^b^Number of patients (percentage)

One hundred and eighty-three patients (37%) received transfusion products (RBC, fresh frozen plasma, platelets and fibrinogen concentrates) during their hospital stay, with RBC being the most common. Most of the transfusions were performed during surgery. All products transfusion and RBC transfusion were significantly higher in the group of iron deficient patients during surgery (respectively 31% vs 19%, *p* = 0.005; and 22% vs 13%, *p* = 0.017), however the incidences during the whole hospital stay were not significantly different between ID and non-ID groups (all products transfusion respectively 42% vs 33%, *p* = 0.07; RBC transfusion respectively 31% vs. 26%, *p* = 0.28). Table 2 summarises transfusion and main outcomes. Blood retransfusion was comparable according to iron status, and there were no significant differences in the main postoperative outcomes between ID and non-ID patients. Eighty-six patients, all anaemic after their surgery, received intravenous iron during the postoperative period.

### Risk factors for RBC transfusion

Variables associated with RBC transfusion, adjusted by centre, are presented in Table 3. Variables independently associated with a lower risk of RBC transfusion were male sex (adjusted OR = 0.23 [0.14–0.39], *p* < 0.001), BMI (adjusted OR = 0.94 [0.89–0.99], *p* = 0.016, per kg m^−2^), and preserved LVEF (adjusted OR = 0.97 [0.95–0.99], *p* = 0.005, per percent of LVEF). Conversely, a severe SAPS II score (adjusted OR = 1.04 [1.02–1.06], *p* = 0.001, per point), reoperation (adjusted OR = 7.26 [1.99–26.39], *p* = 0.003), and combined surgery as compared with isolated valve surgery (adjusted OR = 2.66 [1.40–5.06], *p* = 0.003) were independently associated with a higher risk. ID was not associated with in-hospital RBC transfusion (adjusted OR = 0.85 [0.53–1.36], *p* = 0.49), while pre-operative anaemia (adjusted OR = 4.16 [2.34–7.35], *p* < 0.001) independently was.

**Table 3:** Risk factors for RBC transfusion (logistic regression stratified by centre, stepwise backward elimination)

	**Univariable**			**Multivariable**		
	**OR**	**CI 95%**	***p* value**	**OR**	**CI 95%**	***P*-value**
**Baseline characteristics**						
** SAPS II** (per point)	1.06	1.03–1.08	< 0.001	1.04	1.02–1.06	**0.001**
** Age** (per year)	1.02	1–1.04	0.031			
** Sex** (male)	0.28	0.19–0.43	< 0.001	0.23	0.14–0.39	** < 0.001**
** BMI** (per kg m^−2^)	0.95	0.91–1	0.031	0.94	0.89–0.99	**0.016**
**Comorbidities**						
** ASA score** (per class)	1.8	1.06–3.05	0.029			
** Smoking**	0.76	0.51–1.13	0.18			
** Hypertension**	1.48	0.97–2.24	0.066	1.53	0.94–2.49	0.09
** Dyslipidaemia**	0.93	0.63–1.38	0.73			
** Arteritis**	1.57	0.83–2.96	0.16			
** Angina**	0.89	0.58–1.36	0.59			
** CRF**	1.98	1.15–3.41	0.014			
** Obstructive lung disease**	1.29	0.72–2.31	0.40			
** Diabetes**	0.88	0.53–1.46	0.62			
** Atrial fibrillation**	1.49	0.84–2.64	0.17			
** NYHA class** (per class)	1.63	1.22–2.18	< 0.001	1.34	0.97–1.87	0.079
** LVEF** (per %)	0.98	0.96–1	0.014	0.97	0.95–0.99	**0.005**
** Pulmonary hypertension**	1.74	1–3.03	0.049			
** Active antiplatelet therapy**	1.13	0.77–1.68	0.53			
**Surgery characteristics**						
** Urgent surgery** (*vs* elective)	1.76	0.97–3.2	0.065			
** Reoperation**	4.85	1.6–14.75	0.005	7.26	1.99–26.39	**0.003**
** Type of surgery**						
** Isolated valve**	REF	REF	REF	REF	REF	REF
** Coronary bypass**	0.91	0.58–1.44	0.69	1.46	0.85–2.51	0.168
** Combined surgery**	1.80	1.04–3.13	0.036	2.66	1.40–5.06	**0.003**
** Others**	2.26	0.93–5.31	0.062	2.64	0.99–7.04	0.053
**Biological status**^a^						
** Serum ferritin < 100 ng ml**^**−1**^	0.86	0.55–1.35	0.51	0.70	0.42–1.16	0.17
** Serum ferritin 100–300 ng ml**^**−1**^** and Tsat < 20%**	1.52	0.99–2.34	0.058	1.11	0.67–1.84	0.69
** Iron deficiency**	1.20	0.81–1.78	0.37	0.85	0.53–1.36	0.49
** Anaemia**	3.96	2.41–6.51	< 0.001	4.16	2.34–7.35	** < 0.001**

*ASA American Society of Anesthesiologists*, *BMI* body mass index, *CPB* cardiopulmonary bypass, *CRF* chronic renal failure defined by a glomerular filtration rate < 60 ml min^−1^ 1.73 m^−2^. *LVEF* left ventricular ejection fraction, *NYHA New York Heart Association* classification for dyspnoea. *OR* odd ratio, *RBC* red blood cells, *SAPS II* Simplified Acute Physiology Score 2, *Tsat* transferrin saturation

^a^Each status was tested alone, one by one, in the multivariable analysis

## Discussion

In this multicentre cohort study that investigates both elective and urgent surgeries, we observed that patients with ID had a higher rate of RBC transfusion during surgery, and a higher rate of any blood product transfusion during hospital stay, as compared with iron-replete patients. However, ID was not independently associated with RBC transfusion during the whole hospital stay.

These results need to be interpreted carefully. First, most of RBC transfusions were performed during surgery and were significantly higher in the ID group (22% vs 13%). Moreover, anaemia was a strong driver of RBC transfusion (adjusted OR = 4.16), with IDA being the main cause of anaemia in our population (*n* = 43, 55% of anaemic patients). This is of importance since ID is a modifiable factor, easily attainable as part of a patient blood management (PBM). Ultimately, we observed very low transfusion rates compared to previous literature [21, 22]. The study might be underpowered and suffers from an insufficient number of patients, as we expected higher RBC transfusion rates. We believe that a focused pre-operative screening of at risk of transfusion-ID patients might lead to targeted iron supplementation prior to cardiac surgery and reduction of anaemia, transfusion, and their associated morbidity.

Anaemia already showed its association with transfusion [14, 16]. There is sufficient evidence to recommend a shared haemoglobin cutoff (< 13 g dl^−1^) for both sexes to define anaemia prior to surgeries with a high risk of blood loss [3] and to alleviate the potential disadvantage of women compared with men with regard to impact and optimisation of preoperative anaemia [23]. Although ID has shown its association with poor outcomes in multiple diseases [2, 4], we found that ID alone, without clinical anaemia, may not be strongly associated with in-hospital transfusion requirements in the cardiac surgery setting. Early stage ID, before the anaemic stage, could be insufficient to affect perioperative transfusion. On the other hand, the long-term impact of lower haemoglobin levels at discharge have not been investigated in cardiac surgery, and poor outcomes associated with early stage ID could be delayed.

We also confirmed that RBC transfusion during surgery depends on sex and body mass index, reflecting the impact of haemodilution during CPB. Other worse conditions represented by altered LVEF, severe SAPS 2 scores, reoperations and combined surgeries, were expected and confirmed risk factors for RBC transfusions. Surprisingly, continuation of antiplatelet therapy (mostly acetylsalicylic acid alone and rare cases of dual antiplatelet therapy) was not associated with RBC transfusion.

ID prevalence in our population (37%) was similar to previous single centre reports in scheduled cardiac surgery [5, 7]. AID defined by ferritin levels < 100 ng ml^−1^ has been observed in 21% of patients before elective cardiac surgery [12]. Appending FID (using Tsat < 20% or low haemoglobin density > 4%) to the definition for ID increases the prevalence at 39% [13] to 47% [20]. It is unclear whether FID and AID affect haemoglobin metabolism similarly, and whether their association with preoperative anaemia and RBC transfusion could be comparable. Focusing on “low iron stores according to expected blood loss” prior to surgery [3], instead of determining an “iron-deficient” or “iron-replete” status, could be more suitable to evaluate the impact of iron stores on perioperative transfusion.

PBM and transfusion practice widely differ between centres [22, 24]. In the study, no preoperative PBM treatment was given to our patients, whereas tranexamic acid and blood salvaging were systematically applied during surgery, as recommended [16]. Decision to transfuse was triggered by the anaesthesiologist in charge of the patient, according to applicable recommendations [16–18]. We presume a prime adherence to restrictive transfusion guidelines as we report very low rates of RBC transfusion. Nevertheless, surgical practice and standards for cardioplegia and CPB were different between our hospitals, as was the decision to administer postoperative intravenous iron in order to achieve cardiac rehabilitation.

The validity of the study results is strengthened by the multicentre design and the amount of participants. We collected a number of preoperative and intraoperative data and we precisely established transfusion rules to avoid confounding factors. However, we could not include all consecutive patients presenting for elective and urgent surgeries, with a potential selection bias. Especially, centre C had important organizational issues and/or lack of preoperative iron status determination and ultimately 18 patients included in the analysis. Moreover, as previously discussed, RBC transfusion rates were lower than expected and it might affect the impact of the study. Finally, postoperative complications have a relatively low incidence in non-emergency cardiac surgery and the study was not designed to show a difference for these outcomes.

## Conclusions

In conclusion, anaemia but not ID was independently associated with in-hospital RBC transfusion in cardiac surgery setting. ID remains the main reason and an easily modifiable risk factor for preoperative anaemia, and was associated with more RBC transfusion in the operating room and with lower haemoglobin levels until discharge. There is a need to better identify outcomes associated with ID and to understand which patients, among those with preoperative ID (AID, FID and/or IDA patients) are at risk of transfusion and would benefit from targeted iron supplementation prior to cardiac surgery.

## Data Availability

The datasets used and/or analysed during the current study are available from the corresponding author on reasonable request.

## Abbreviations

AID: Absolute iron deficiency
AKF: Acute kidney failure
ASA: *American Society of Anesthesiologists*
BMI: Body mass index
CPB: Cardiopulmonary bypass
CRF: Chronic renal failure
FID: Functional iron deficiency
Hb: Hemoglobin
ICU: Intensive care unit
ID: Iron deficiency
IDA: Iron deficiency anemia
LVEF: Left ventricular ejection fraction
NYHA: *New York Heart Association* Classification for dyspnea
OR: Odd ratio
PO: Postoperative
RBC: Red blood cell
SAPS II: Simplified Acute Physiology Score 2
Tsat: Transferrin saturation
